# CircRNA WHSC1 targets the miR‐646/NPM1 pathway to promote the development of endometrial cancer

**DOI:** 10.1111/jcmm.15346

**Published:** 2020-05-07

**Authors:** Yao Liu, Shuo Chen, Zhi‐Hong Zong, Xue Guan, Yang Zhao

**Affiliations:** ^1^ Department of Gynecologic Oncology Research Office The Third Affiliated Hospital of Guangzhou Medical University Guangzhou China; ^2^ Department of Gynecology The Third Affiliated Hospital of Guangzhou Medical University Guangzhou China; ^3^ Key Laboratory for Major Obstetric Diseases of Guangdong Province Key Laboratory of Reproduction and Genetics of Guangdong Higher Education Institute in Guangdong Province Guangzhou China; ^4^ Department of Gynecology The First Affiliated Hospital of China Medical University Shenyang China

**Keywords:** circular RNA, circWHSC1, endometrial cancer, microRNA, NPM1

## Abstract

Circular RNAs (circRNAs) play important roles in human cancer progression. Their high stability and tissue specificity make circRNAs important molecular targets for clinical diagnosis, treatment and prognosis. However, the functions and molecular mechanisms of circRNA WHSC1 in endometrial cancer are unknown. CircWHSC1 expression in normal endometrial and endometrial cancer tissues was detected using PCR. Overexpression or knockdown of circWHSC1 in endometrial cancer cell lines HEC‐1B or Ishikawa, respectively, cell function experiments were used to detect the impact of circWHSC1 on endometrial cancer cells. A nude mouse xenograft model was used to detect changes in tumorigenesis of HEC‐1B cells after circWHSC1 overexpression. Bioinformatics and dual luciferase reporter gene technology were used to predict and validate the sponging ability of circWHSC1 on microRNAs. Gene expression changes were detected by using Western blotting. CircWHSC1 expression was increased in endometrial cancer tissues. CircWHSC1 overexpression promoted the proliferation, migration and invasion of endometrial cancer cells and decreased apoptosis. CircWHSC1 knockdown had the opposite effect. CircWHSC1 overexpressed nude mice showed increased tumorigenicity. Bioinformatics predicted that circWHSC1 binds to miR‐646, which was confirmed using luciferase reporter gene assays. High expression of miR‐646 could reverse the effect of circWHSC1 on endometrial cancer cells. Western blotting showed increased or decreased levels of nucleophosmin 1 (NPM1), an miR‐646 downstream target, after circWHSC1 overexpression or knockdown, respectively. CircWHSC1 promotes endometrial cancer development through sponging miR‐646 and targeting NPM1.

**Abbreviations**
CircRNA: circular RNA
miRNA: microRNA
MTT: 3‐(4,5‐dimethylthiazol‐2‐yl)‐2,5‐diphenyltetrazolium bromide
NPM1: nucleophosmin 1
qRT‐PCR: quantitative real‐time reverse transcription PCR

## 1 INTRODUCTION

Endometrial cancer is the most common gynaecological malignancy, and its incidence has increased rapidly over the past decade.^1^ Currently, early diagnosis of endometrial cancer is associated with good prognosis; however, for asymptomatic women, there is no good diagnostic screening method.^2^ Therefore, it is urgent to explore the mechanism of endometrial cancer development and to identify biomarkers for diagnosis, treatment and prognosis.

Circular RNAs (circRNAs) are a new class of endogenous non‐coding RNAs that form a covalently closed structure by reverse splicing at their 3′ and 5′ ends.^3^ CircRNAs are stable, abundant and conserved. They are usually expressed in specific tissues or specific developmental stages and may be derived from exons, introns or intergenic regions. CircRNAs regulate gene expression (especially parental genes) through scaffolds assembled as protein complexes. CircRNAs also regulate alternative splicing and RNA‐protein interactions, as well as sponging microRNAs (miRNAs).^4^ There is increasing evidence that circRNAs play a crucial regulatory role in different types of tumours, including hepatocellular carcinoma, breast cancer, gastric cancer, pancreatic cancer, osteosarcoma and thyroid cancer, suggesting that circRNAs can act as oncogenes, and are potential biomarkers and therapeutic targets.^5^ However, there are few studies on circRNAs and endometrial cancer, and the function of circRNAs in endometrial cancer remains unknown. We identified a circRNA WHSC1, which was highly expressed in ovarian cancer and promoted the development of ovarian cancer according to our previous studies,^6^ but its role in endometrial cancer is unclear. So the present study explored the role of circWHSC1 in endometrial cancer and its possible molecular mechanisms.

## 2 MATERIALS AND METHODS

### 2.1 Tissue specimens

All specimens were obtained from the Department of Obstetrics and Gynecology, the First Affiliated Hospital of China Medical University. A total of 26 normal endometrial tissues and 32 endometrial cancer tissues were confirmed pathologically. The protocol was approved by the ethical review committee of our institution, and patient consent was obtained before the samples were taken.

### 2.2 Cell line culture and transfection

The human endometrial cancer cell lines HEC‐1B and Ishikawa were cultured in high glucose DMEM and RPMI‐1640 medium containing 1% streptomycin and 10% foetal bovine serum, and cultured in an incubator. The cells were exchanged or passaged according to the amount of cells. Transfection was performed using Lipofectamine™ 3000.

### 2.3 Cell proliferation assay

Cells in the log phase were digested with trypsin, and a cell suspension was prepared. Cells at 3000 per well were added to a 96‐well plate and cultured by simultaneously adding 100 µL of the culture solution. The experiment was divided into two groups: the negative control group transfected with vector or sh‐NC, and the treatment group transfected with circWHSC1 or sh‐circWHSC1, and cell proliferation was recorded at 0, 24, 48 and 72 hours. At each time point, 20 µL (5 mg/mL) of pre‐configured MTT reagent was added and incubated for 2 hours in a 37°C constant temperature. The cell culture medium was then removed, 150 µL of dimethyl sulfoxide was added, and the cells were incubated in the dark, with low speed shaking. Then, the absorbance value at 490 nm was read using a microplate reader.

### 2.4 Apoptosis assay

HEC‐1B and Ishikawa cells were seeded at a density of 300 000 cells per well in a 6‐well culture plate and transfected circWHSC1 or sh‐circWHSC1 after the cells had attached. After culturing for 48 hours, the cells were collected, and the apoptosis rate was analysed using a flow cytometer according to the instructions of the Annexin VPE/7AAD Apoptosis Detection Kit (BD Biosciences, San Jose, CA, USA).

### 2.5 Cell scratch assay

The experimental group and the control group cells were inoculated into wells of culture plates and grown to more than 80% confluence. The cell layer was scratched with a fine line, washed, and 2 mL of serum‐free medium was added to each well. The cells were then cultured in a 37°C incubator, and the scratch widths were imaged under an inverted microscope at 0 and 48 hours, respectively. Cell mobility was expressed as the percentage of the distance travelled compared with the initial width of the scratch.

### 2.6 Cell invasion assay

After 50 mg/L of Matrigel was diluted at a ratio of 1:15, about 32 µL was added to the upper chamber of the bottom membrane of a transwell chamber (BD Bioscience, USA) to uniformly cover the upper chamber surface of the membrane. The cells were then digested, and the concentration was calculated; then, 600 µL of the serum‐containing culture solution was added to the lower chamber of the 24‐well plate. Serum‐free cell suspension containing 5 × 10^4^ cells (200 µL) was added to the upper chamber. The cells were then incubated for 48 hours to remove non‐invasive metastatic cells on the stromal membrane. After fixation, staining and drying, the cells that migrated into the lower chamber were placed on a slide, and the number of migrating cells was counted under a microscope.

### 2.7 In vivo tumour formation experiment

Female nude mice were purchased from Vital River Laboratories (Beijing, China) and reared in specific pathogen‐free environment of the department of Laboratory Animal science of china medical university (Shenyang, China). The animal experiment was approved by the Ethics Review Committee of the department of Laboratory Animal science of china medical university (IACUC Issue No: 2018145). HEC‐1B cells (1 × 10^7^) transfected with circWHSC1 (or vector‐transfected) suspended in PBS were injected subcutaneously into the mice. The tumour volume was measured twice a week until it could be observed visually. After 4 weeks, the mice were sacrificed and the tumour tissue was excised and measured.

### 2.8 Quantitative real‐time reverse transcription PCR

Tissue samples and cells were collected, and total RNA was extracted using the TRIzol reagent (Invitrogen, Waltham, MA, USA) for quantitative real‐time reverse transcription PCR (qRT‐PCR) detection. The cDNA obtained by reverse transcription using the Promega GoScript reverse transcription system (Promega Madison, WI, USA) was subjected to quantitative real‐time PCR in a 20 µL PCR amplification system using the Light Cycle 480 real‐time quantitative PCR instrument. The differences in the expression levels of circWHSC1 were analysed using 2^−ΔΔCt^ relative quantification.

### 2.9 Western blotting

Total proteins were extracted from tissue and cells, and the proteins in the sample were separated using SDS‐polyacrylamide gel electrophoresis. The separated proteins were then transferred to a polyvinylidene fluoride (PVDF) membrane. The PVDF membrane was immersed in blocking solution and shaken at room temperature for 1 hour. Then, the membrane was incubated with anti‐NMP1 primary antibodies(1:1000; Proteintech, Rosemont, IL, USA) for 2 hours at room temperature. Then, the membrane was incubated with secondary antibodies [horseradish oxidase‐labelled goat anti‐rabbit IgG (H + L); 1:5000] and was shaken at room temperature for 1 hour. After washing, the membrane was incubated with the developer solution and exposed to an X‐ray film to visualize the immunoreactive proteins.

### 2.10 Dual luciferase reporter gene assay

The bioinformatics website (https://circinteractome.nia.nih.gov/) was used to predict possible miRNA binding sites for circWHSC1. Then luciferase reporter genes for circWHSC1 and the predicted miRNAs were then constructed. Adherent HEK293T cells were transfected with the wild‐type circWHSC1 or mutant control clones (vector psiCHECK2) and transfected with miRNA mimics or negative controls. After 48 hours of culture, the firefly fluorescence values and the Renilla fluorescence values were measured, and the results were expressed as the ratio of the two fluorescence values.

### 2.11 Statistical analysis

Statistical analysis was performed using GraphPad Prism 5 software (GraphPad Software, Inc, La Jolla, CA, USA). All functional experimental values are described as the mean ± SD values and were repeated three times. *P* value < 0.05 was considered statistically significant.

## 3 RESULTS

### 3.1 Expression of circWHSC1 in endometrial carcinoma tissues and cells

**FIGURE 1:**
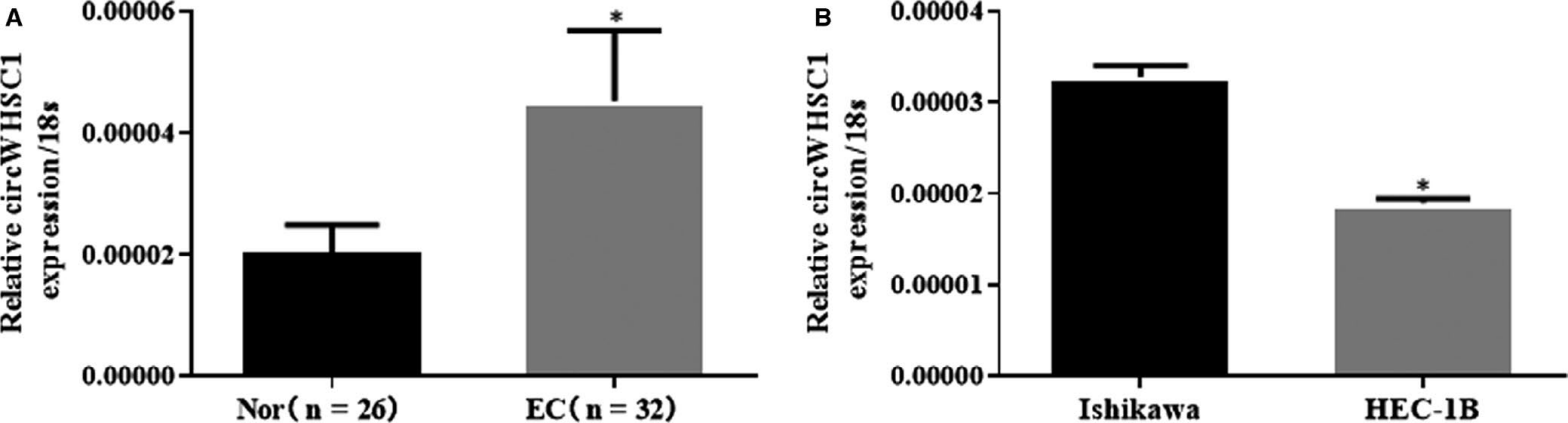
Expression of circWHSC1 in endometrial carcinoma tissues and cells. CircWHSC1 was significantly higher in endometrial cancer than in normal endometrial tissue (A). Ishikawa cells expressed higher levels of circWHSC1 than HEC‐1B cells (B); the results are representative of three separate experiments. *P < 0.05

The expression levels of circWHSC1 in 26 normal endometrium and 32 endometrial cancer specimens were detected using PCR. The results showed that circWHSC1 was highly expressed in cancer tissues compared with that in the normal tissues (Figure 1A, *P < 0.05) (details could be found in Tables S1 and S2). The expression of circWHSC1 was detected in endometrial cancer cell lines HEC‐1B and Ishikawa. The results showed that Ishikawa cells expressed higher levels of circWHSC1 than HEC‐1B cells (Figure 1B, *P < 0.05).

### 3.2 CircWHSC1 promotes the proliferation of endometrial cancer and inhibits apoptosis

**FIGURE 2:**
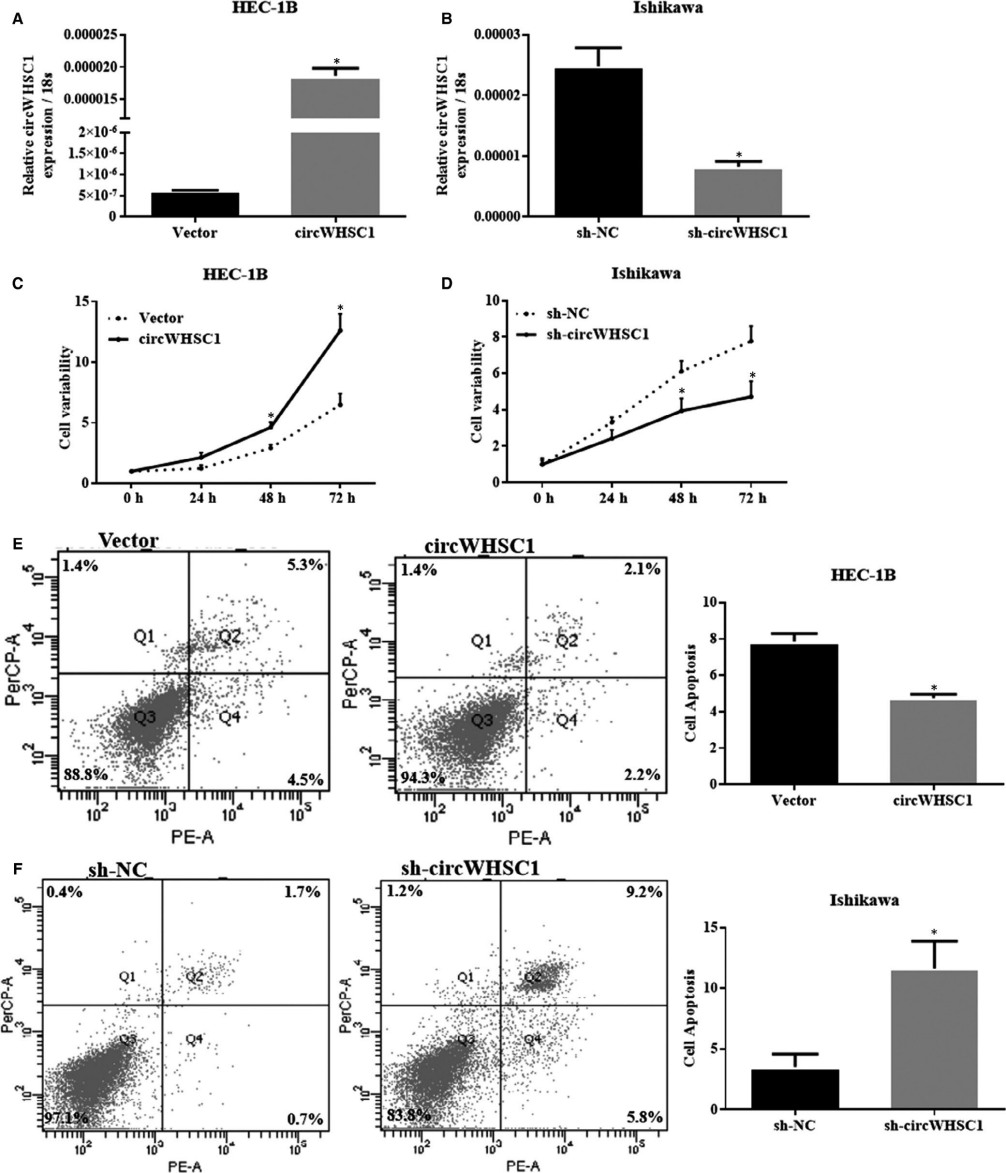
CircWHSC1 promotes proliferation and inhibits apoptosis of endometrial cancer cells. After circWHSC1 transfection, HEC‐1B cell exhibited significantly higher circWHSC1 expression (A); silencing of circWHSC1 resulted in lower circWHSC1 expression in Ishikawa (B). The up‐regulation of circWHSC1 expression increased cell proliferation (C) and decreased apoptosis (E) in HEC‐1B cells; silencing of circWHSC1 resulted in opposite effect in Ishikawa cells (D and F). The results are representative of three separate experiments; *P < 0.05

DNA encoding circWHSC1 was transfected into HEC‐1B (low circWHSC1 expression) for overexpression, and a short hairpin RNA (sh‐circWHSC1) was transfected into Ishikawa cells (high circWHSC1 expression) to knockdown circWHSC1 (details of the sequences could be found in Table S3). The transfection efficiency was detected by PCR (Figure 2A,B, *P < 0.05). Cell proliferation and apoptosis experiments demonstrated that overexpression of circWHSC1 promoted cell proliferation and inhibited apoptosis (Figure 2C,E, *P < 0.05), whereas sh‐circWHSC1 inhibited cell proliferation and promoted apoptosis (Figure 2D,F, *P < 0.05).

### 3.3 CircWHSC1 promotes the migration and invasion of endometrial cancer cells

**FIGURE 3:**
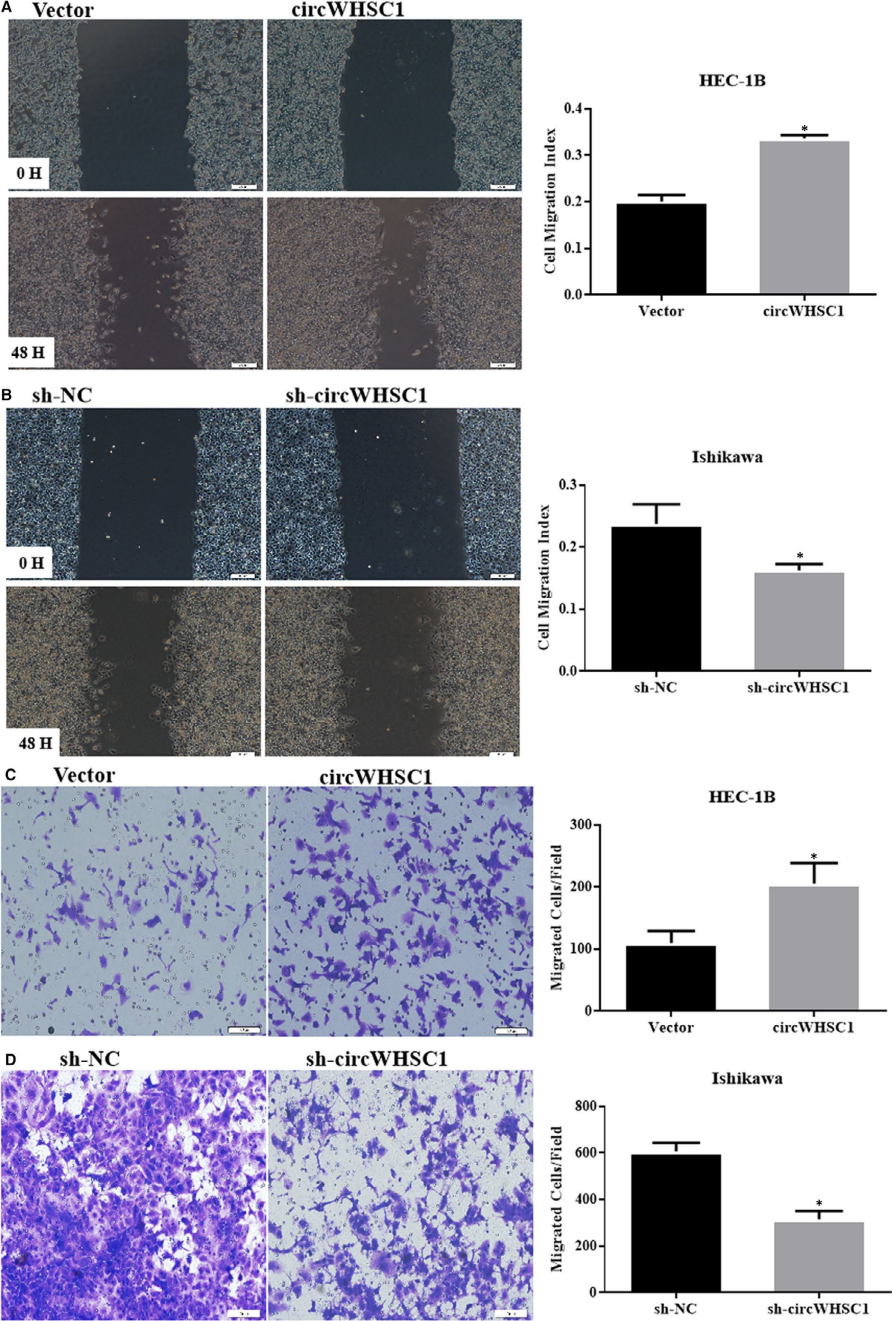
CircWHSC1 promotes the migration and invasion of endometrial cancer carcinoma. Up‐regulation of circWHSC1 expression increased cell migration (A) and invasion (C) in HEC‐1B cells. Silencing of circWHSC1 resulted in opposite effect in Ishikawa cells (B and D). The results are representative of three separate experiments; *P < 0.05

Cell migration and cell invasion experiments demonstrated that high expression of circWHSC1 promoted the migration and invasion of HEC‐1B cells (Figure 3A,C, *P < 0.05), and knockdown of circWHSC1 inhibited the migration and invasion of Ishikawa cells (Figure 3B,D, *P < 0.05).

### 3.4 CircWHSC1 promotes tumour growth in vivo

**FIGURE 4:**
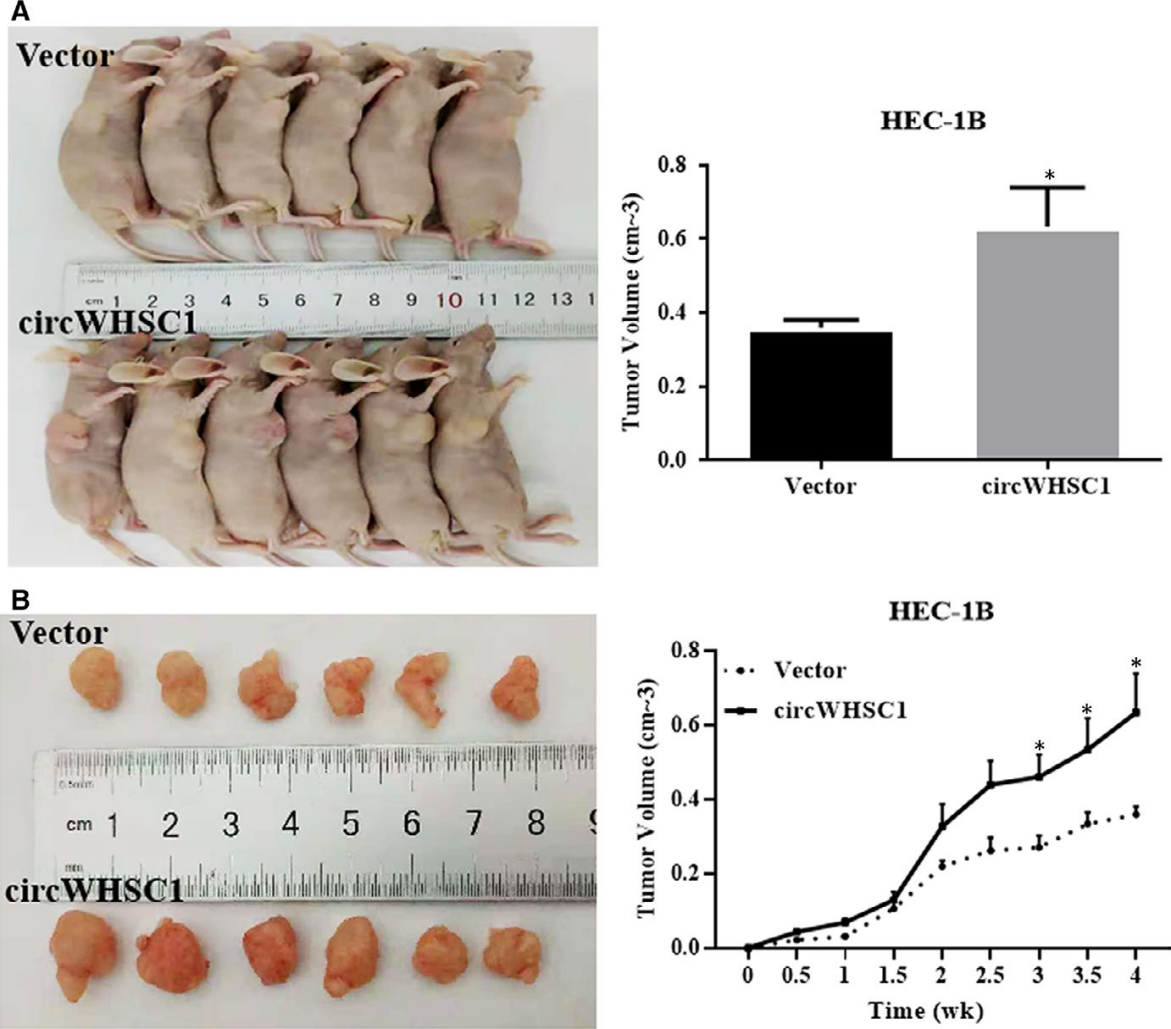
Effect of circWHSC1 on in vivo tumour growth. Overexpression of circWHSC1 in HEC‐1B had a significantly greater tumour formation volume than that in the control group which transfected with vector (A‐D). *P < 0.05

In vivo tumour formation experiments demonstrated that overexpression of circWHSC1 promoted the growth of HEC‐1B‐derived tumours in nude mice compared with that in the control group comprising tumours derived from HEC‐1B cells transfected with the empty vector (Figure 4A‐D, *P < 0.05).

### 3.5 CircWHSC1 targets the miR‐646/nucleophosmin 1 pathway to promote endometrial cancer development

**FIGURE 5:**
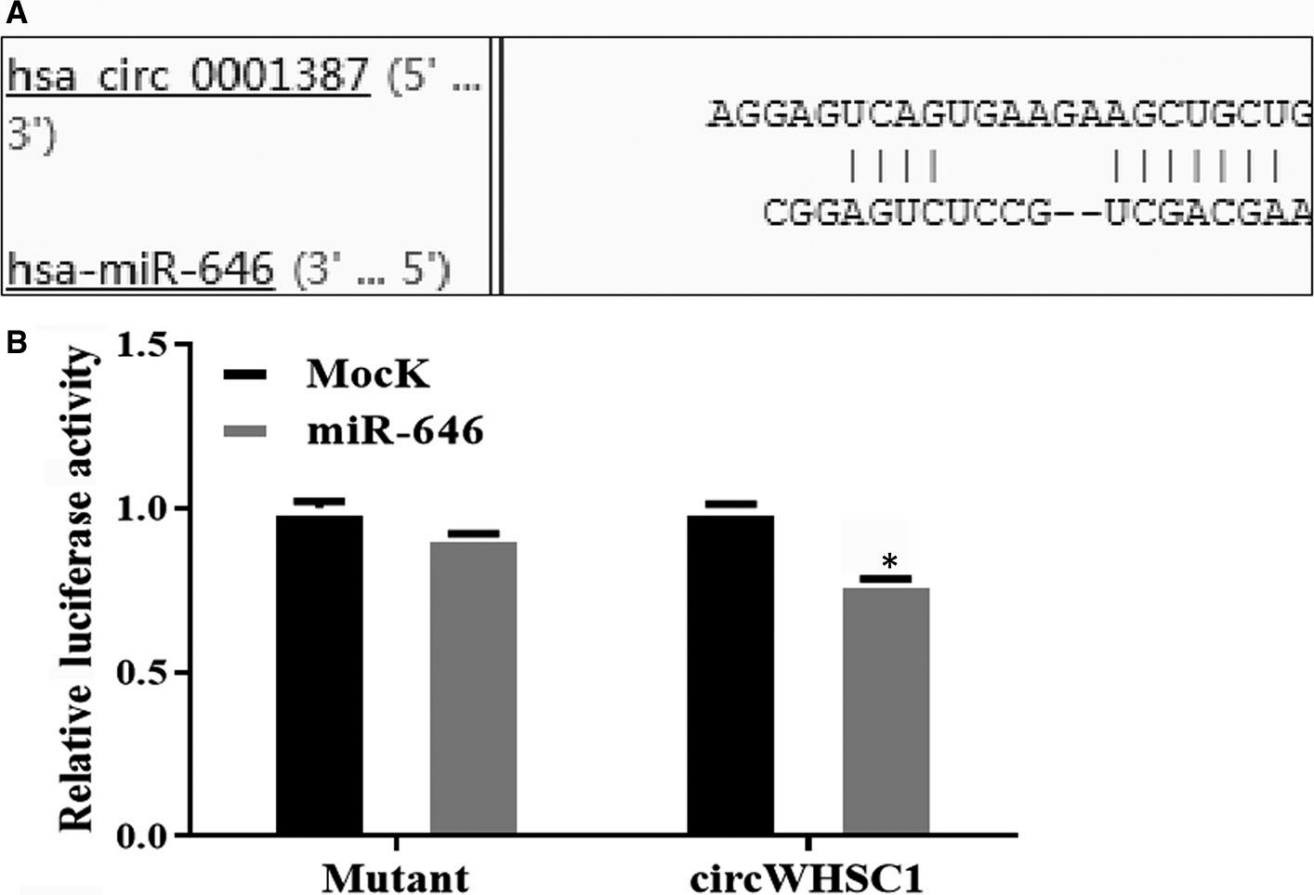
CircWHSC1 can be directly combined with miR‐646. Bioinformatics software predicted the presence of a binding site for circWHSC1 on miR‐646 (A). circWHSC1 bound directly to miR‐646 in HEK293 cells (B)

**FIGURE 6:**
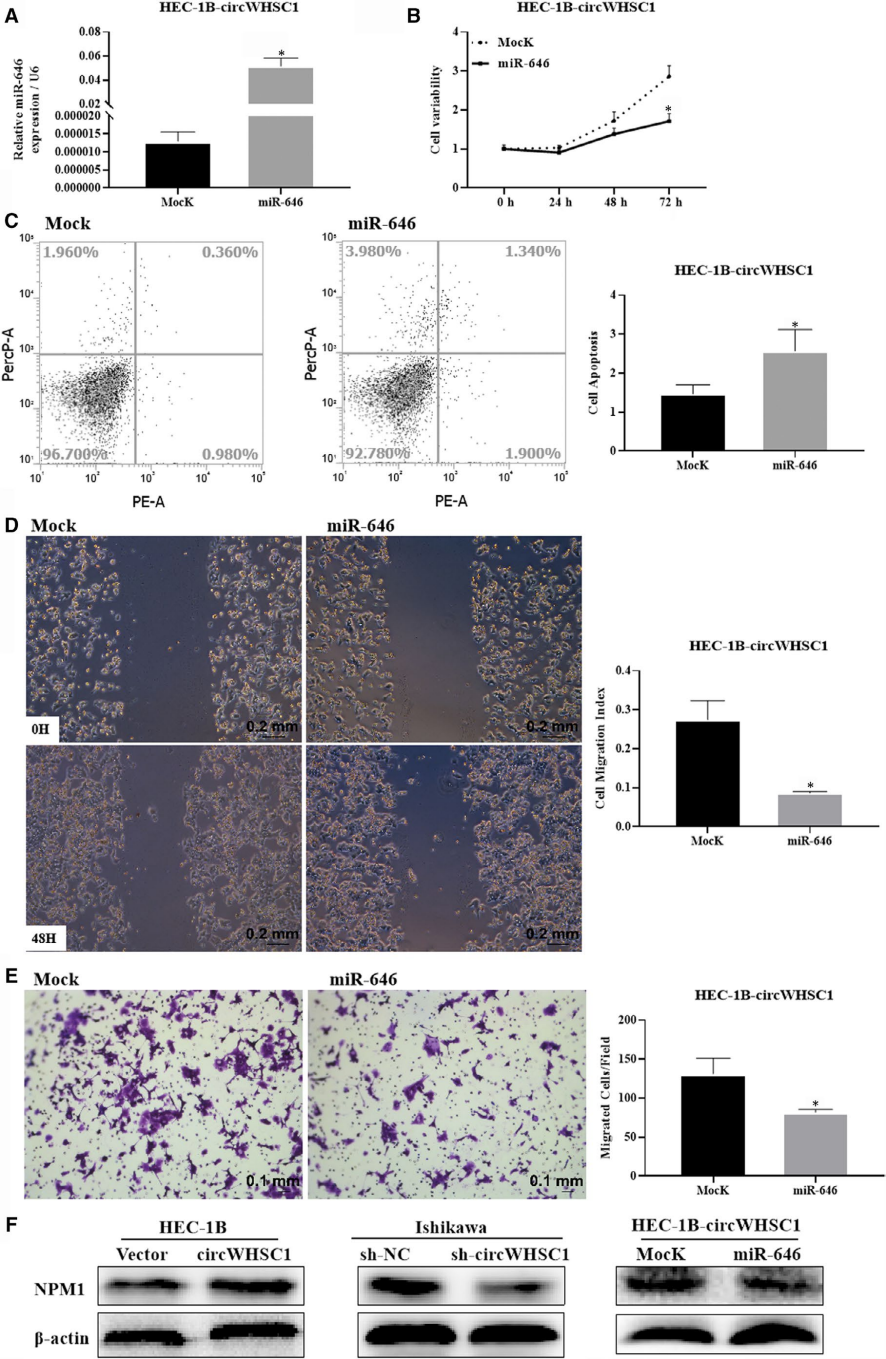
CircWHSC1 targets the miR‐646‐NPM1 pathway to promote endometrial cancer development. The expression of miR‐646 increased significantly after miR‐646 was overexpressed in HEC‐1B overexpressing circWHSC1 cells (A). MiR‐646 reduced cell proliferation (B), migration (D) and invasion (E), and promotes apoptosis (C) in HEC‐1B overexpressing circWHSC1 cells. Overexpression of circWHSC1 increased the expression of NPM1, and vice versa, whereas miR‐646 overexpression reduces NPM1 expression in HEC‐1B overexpressing circWHSC1 cells (F). The results are representative of three separate experiments. *P < 0.05. NPM1, nucleophosmin 1m

We predicted the miRNAs that might bind to circWHSC1 using the bioinformatics website (https://circinteractome.nia.nih.gov/) and found that circWHSC1 has a binding site for miR‐646. Dual luciferase reporter gene assays confirmed that circWHSC1 binds directly to miR‐646 (Figure 5A,B, *P < 0.05). Then, we overexpressed miR‐646 in HEC‐1B cells that up‐regulated circWHSC1 (Figure 6A, *P < 0.05), and detected the changes in cell phenotype. It was found that miR‐646 can reverse the effects of circWHSC1 on promoting cell proliferation, migration and invasion (Figure 6B,D,E, *P < 0.05). At the same time, overexpression of miR‐646 can promote cell apoptosis (Figure 6C, *P < 0.05).

It has been reported that miR‐646 binds to the mRNA encoding nucleophosmin 1 (NPM1) in endometrial cancer and exerts a tumour suppressor effect.^7^ We examined the effect of circWHSC1 on the levels of downstream protein NPM1 of miR‐646 using Western blotting and found that overexpression of circWHSC1 promoted the levels of NPM1, and silencing circWHSC1 decreased the levels of NPM1. Meanwhile, overexpression of miR‐646 can reverse the effect of circWHSC1 on promoting NPM1 expression (Figure 6F). Therefore, circWHSC1 targets the miR‐646/NPM1 pathway to exert its role in tumour development.

## 4 DISCUSSION

Circular RNAs were originally thought to be by‐products of aberrant splicing and did not attract much attention. However, the development of bioinformatics and high‐throughput sequencing technologies has led to the identification of widespread circRNA expression in different cell types and species. A growing number of studies have shown that circRNAs are differentially expressed in a variety of cancers and are involved in various cellular activities, including proliferation, differentiation, invasion and metastasis, and have a strong relationship with disease status and prognosis.^8^, ^9^ For example, circRNAs *EXOC6B* and *N4BP2L2* can be used as prognostic biomarkers for epithelial ovarian cancer, and circ_0081001 is a potential marker for the diagnosis of osteosarcoma.^10^, ^11^ In the present study, we identified a circRNA, circWHSC1, which is highly expressed in endometrial cancer tissues and was hypothesized to play a role in the development of endometrial cancer.

To explore the function of circWHSC1 in endometrial cancer, its expression was up‐regulated in HEC‐1B cells, which express a relatively low level of circWHSC1. Overexpression of circWHSC1 promoted cell proliferation, migration and invasion, and inhibited apoptosis. By contrast, Ishikawa cells, which express a relatively high level of circWHSC1, were subjected to circWHSC1 down‐regulation, which inhibited cell proliferation, migration and invasion, and promoted apoptosis. Tumour formation in nude mice showed that circRNA circWHSC1 could promote tumour growth in vivo. Therefore, circWHSC1 plays a cancer‐promoting role in endometrial cancer cell lines.

MicroRNAs are small, non‐coding single‐stranded RNAs that, by binding to the 3'‐untranslated region (3'‐UTR) of mRNAs, induce mRNA degradation or inhibit protein translation.^12^, ^13^ MiRNAs are involved in the regulation of tissue homeostasis and the pathogenesis of human diseases. Functional studies have confirmed that miRNAs are intimately involved in the development, proliferation, metastasis and invasion of tumour cells.^14^, ^15^, ^16^

Studies have shown that certain circRNAs are extremely rich in miRNA binding sites and are involved in the development of cancer by adsorbing miRNAs, acting as an miRNA sponge.^17^ For example, circ‐*ZEB1.33* is highly expressed in hepatocellular carcinoma tissues, and by sponging miR‐200a‐3p, up‐regulates CDK6 levels, leading to the promotion of hepatocellular carcinoma proliferation.^18^ In bladder cancer, overexpression of circ‐*VANGL1* promotes cancer development by regulating the miR‐605‐3p‐VANGL1 pathway.^19^ More importantly, the analysis of circRNA as competitive endogenous RNAs has been reported in cancers such as colon cancer, ovarian cancer and breast cancer.^20^, ^21^, ^22^

Therefore, we sought to identify miRNAs that bind to circWHSC1. Bioinformatic analysis predicted that circWHSC1 has binding sites for miRNA‐646. We verified that circWHSC1 could bind to miRNA‐646 using luciferase reporter gene assays. miRNA‐646 has been reported to be down‐regulated in many human cancers and is involved in the development of many malignant tumours as a tumour suppressor gene, including colon cancer, pancreatic cancer, gastric cancer, lung cancer, osteosarcoma and clear cell kidney cancer.^23^, ^24^, ^25^, ^26^, ^27^, ^28^ In endometrial cancer, miR‐646 inhibits cell proliferation, migration and invasion, and targets NPM1 mRNA to negatively regulate the progression of endometrial cancer.^7^

Nucleophosmin 1 is a high‐level nucleolar phosphoprotein in the nucleolar granules and is a multifunctional protein involved in various cellular activities, including the transport of pre‐ribosomal particles and ribosome biosynthesis. Body replication, response to stress stimuli, regulation of DNA transcription, maintenance of genomic stability and embryonic development play a crucial role in tumorigenesis.^29^ Dysregulation of NPM1 has been observed in various human cancers in which it promotes or inhibits the development and progression of cancer.^30^, ^31^, ^32^ NPM1 is highly expressed in endometrial cancer and correlates positively with the clinical stage and histological grade of endometrial cancer.^7^ Our Western blotting analysis showed that overexpression of circWHSC1 promoted NPM1 expression, although down‐regulation of circWHSC1 inhibited NPM1 expression. At the same time, miR‐646 overexpression can reverse the effect of circWHSC1 on promoting NPM1 expression.

Therefore, we propose that circWHSC1 promotes the occurrence and development of endometrial cancer by the sponging miRNA‐646, thereby increasing the level of NPM1.

## 5 CONCLUSION

Our study was the first to find that circWHSC1 was overexpressed in endometrial carcinoma than normal endometrial tissue. We discovered for the first time that circWHSC1 binds directly to miR‐646 and regulated the expression of NPM1. We supposed that targeting of circWHSC1 expression may prove to be a novel insight for early diagnosis and genetic therapeutic strategy for endometrial carcinoma.

## CONFLICT OF INTERESTS

The authors have no conflicts of interest to declare.

## DECLARATIONS

The research protocol was approved by the China Medical University Ethics Committee (No: 2016‐32‐2).

## CONSENT FOR PUBLICATION

Not applicable.

## Supporting information

Table S1‐S3

## Data Availability

The data sets used and/or analysed during the current study available from the corresponding author on reasonable request.
