# Supplementary material for: CircRNA WHSC1 targets the miR‐646/NPM1 pathway to promote the development of endometrial cancer
Source: J Cell Mol Med. 2020 May 7;24(12):6898–907. doi: 10.1111/jcmm.15346 (PMC7299690; doi:10.1111/jcmm.15346)
Supplement: Supplementary file 1 — Table S1‐S3 [file JCMM-24-6898-s001.doc]

**Supplementary Table 1:** circRNA WHSC1 expression in normal Normal endometrium and Endometrial cancer tissues

| **Groups** | **N** | ***WHSC1* expression / 18s** | ***P* value** |
| --- | --- | --- | --- |
|
| Normal endometrium | 26 | 2.14E-05 ±1.8E-05 | ***0.029*** |
| Endometrial cancer | 32 | 4.52E-05 ±6.56E-05 |  |

Bold and Italics means P < 0.05.

**Supplementary Table 2:** Correlation of WHSC1 expression with different clinicopathological features of Endometrial cancer

| **Clinicopathological features** | **N** | ***WHSC1* expression / 18s** | ***P* value** |
| --- | --- | --- | --- |
|
| The depth of myometrial infiltration |  |  | ***0.027*** |
| < 1/2 | 29 | 4.77E-05 ±6.85E-05 |  |
| ≥ 1/2 | 3 | 2.16E-05 ±4.56E-06 |  |
| **Age** |  |  | 0.187 |
| ≤55 | 16 | 2.98E-05 ±1.75E-05 |  |
| > 55 | 16 | 6.07E-05 ±8.99E-05 |  |
| **FIGO stages** |  |  | 0.307 |
| I | 28 | 4.02E-05 ±5.01E-05 |  |
| II-IV | 4 | 8.02E-05 ±1.41E-04 |  |
| **Pathology classification** |  |  | 0.412 |
| Well | 14 | 3.65E-05±5.62E-05 |  |
| Mod +Poor | 18 | 8.32E-05±9.36E-05 |  |
| Bold and Italics means P < 0.05. | | | |

**Supplementary Table 3:** The sequence of circWHSC1 is TGTTCTAAGAACGGAAGCATCTGGGCTGGATGGAATTTAGCATCAAGCAGAGTCCCCTTTCTGTTCAGAGTGTTGTAAAGTGCATAAAGATGAAGCAGGCACCAGAAATCCTCGGCAGTGCCAACGGGAAGACTCCGAGCTGCGAGGTGAACCGCGAGTGTTCTGTGTTCCTCAGCAAAGCCCAGCTCTCCAGTAGCCTGCAGGAGGGGGTCATGCAGAAGTTTAACGGCCACGACGCCCTGCCCTTTATTCCAGCCGACAAGCTGAAAGATCTTACTTCCCGGGTGTTTAATGGAGAACCCGGCGCACACGATGCCAAACTGCGTTTTGAGTCCCAGGAAATGAAAGGGATTGGGACACCCCCTAACACTACCCCTATCAAAAATGGCTCTCCAGAAATTAAGCTGAAAATCACCAAAACATACATGAATGGGAAGCCTCTCTTTGAATCTTCCATTTGTGGTGACAGTGCTGCTGATGTGTCTCAGTCAGAAGAAAATGGACAAAAACCAGAAAACAAGGCGAGAAGGAACAGGAAGAGGAGCATAAAATATGACTCCTTGCTGGAGCAGGGCCTTGTCGAAGCAGCTCTTGTGTCTAAGATCTCAAGTCCTTCAGATAAAAAGATTCCAGCTAAGAAAGAGTCTTGTCCAAACACTGGAAGAGACAAAGACCACCTGTTGAAATACAACGTTGGTGATTTGGTGTGGTCCAAAGTGTCGGGTTACCCTTGGTGGCCTTGCATGGTTTCTGCAGATCCACTCCTTCACAGCTATACCAAACTTAAAGGTCAGAAAAAGAGTGCACGCCAGTATCACGTACAGTTCTTTGGTGACGCCCCAGAAAGAGCTTGGATATTTGAGAAGAGCCTCGTAGCTTTTGAAGGAGAAGGACAGTTTGAAAAATTATGCCAGGAAAGTGCCAAGCAGGCACCCACGAAAGCTGAGAAAATTAAGCTATTGAAACCAATTTCAGGGAAATTGAGGGCCCAGTGGGAAATGGGCATTGTTCAAGCAGAAGAAGCTGCAAGCATGTCAGTGGAGGAGCGGAAAGCCAAGTTCACCTTTCTCTATGTGGGGGACCAGCTTCATCTCAACCCTCAAGTAGCCAAGGAGGCTGGCATTGCTGCAGAGTCTTTGGGAGAAATGGCAGAATCCTCAGGAGTCAGTGAAGAAGCTGCTGAAAACCCCAAGTCTGTGAGAGAAGAGTGCATTCCCATGAAGAGAAGGCGGAGGGCCAAACTGTGTAGCTCTGCAGAGACCCTGGAGAGTCACCCCGACATAGGGAAGAGTACTCCTCAAAAGACGGCAGAGGCTGACCCCAGAAGAGGAGTAGGGTCTCCTCCTGGGAGGAAGAAGACCACAGTCTCCATGCCACGAAGCAGGAAGGGAGATGCAGCATCCCAGTTTTTGGTCTTCTGTCAAAAACACAGGGATGAGGTGGTAGCTGAGCACCCAGATGCTTCAGGTGAGGAGATTGAAGAGCTGCTCAGGTCACAGTGGAGTCTGCTGAGTGAGAAGCAGAGAGCACGCTACAACACCAAGTTTGCCCTGGTGGCCCCTGTCCAGGCTGAAGAAGACTCTGGTAATGTAAATGGGAAAAAAAGAAACCACACAAAGAGGATACAGGACCCTACAGAAGATGCTGAAGCTGAGGACACACCCAGGAAAAGACTCAGGACGGACAAGCACAGTCTTCGGAAG.

The sequences of shRNA targeting circRNA *WHSC1* are:

Top strand:

GATCCGAGTCTTCGGAAGTGTTCTAAGAACTTCAAGAGAGTTCTTAGAACACTTCCGAAGACTTTTTTTC

Bottom strand:

AATTGAAAAAAAGTCTTCGGAAGTGTTCTAAGAACTCTCTTGAAGTTCTTAGAACACTTCCGAAGACTCG
